# Using the Intrinsic Geometry of Binodal Curves to Simplify the Computation of Ternary Liquid–Liquid Phase Diagrams

**DOI:** 10.3390/e25091329

**Published:** 2023-09-13

**Authors:** Nataliya Shcherbakova, Vincent Gerbaud, Kevin Roger

**Affiliations:** Laboratoire de Génie Chimique, Université de Toulouse, CNRS, INP, UPS, 31432 Toulouse, France; vincent.gerbaud@ensiacet.fr (V.G.); kevin.roger@cnrs.fr (K.R.)

**Keywords:** LLE ternary diagrams, binodal curves, spinodal curves, differential path-following method, ordinary differential equations

## Abstract

Phase diagrams are powerful tools to understand the multi-scale behaviour of complex systems. Yet, their determination requires in practice both experiments and computations, which quickly becomes a daunting task. Here, we propose a geometrical approach to simplify the numerical computation of liquid–liquid ternary phase diagrams. We show that using the intrinsic geometry of the binodal curve, it is possible to formulate the problem as a simple set of ordinary differential equations in an extended 4D space. Consequently, if the thermodynamic potential, such as Gibbs free energy, is known from an experimental data set, the whole phase diagram, including the spinodal curve, can be easily computed. We showcase this approach on four ternary liquid–liquid diagrams, with different topological properties, using a modified Flory–Huggins model. We demonstrate that our method leads to similar or better results comparing those obtained with other methods, but with a much simpler procedure. Acknowledging and using the intrinsic geometry of phase diagrams thus appears as a promising way to further develop the computation of multiphase diagrams.

## 1. Introduction

Equilibrium phase diagrams testify how a complex meso/macroscopic behaviour emerges from a diversity of intermolecular interactions. Their knowledge thus guides us into unravelling the multi-scale relationship that underlies the properties of a given mixture, whether at equilibrium or during a dynamic process, for instance, liquid–liquid separation. In practice, gaining such knowledge requires either a straightforward computation from the prior knowledge of the thermodynamic potential (direct problem), or the determination of such a potential (inverse problem), usually the Gibbs free energy for liquid mixtures, from the experimental results. These direct and inverse problems are interrelated, as it is, in reality, impossible to obtain the expression of the thermodynamic potential without any prior experimental knowledge. Both experiments and computations are thus required to obtain phase diagrams. While this topic has received a lot of attention, due to its central role, it strikingly remains challenging nowadays to effectively compute phase diagrams from an experimental data set. Fast and reliable algorithms are needed to solve the computational problems arising such as the identification of model parameters from the experimental data, the localization of phase separation envelopes, and the detection of multi-phase regions. A vast literature points out the trial of treating even the common case of ternary systems that can undergo phase separation into two phases.

This observation is striking since the key features of the phase diagram of a common ternary mixture are well known and can be considered textbook material, for instance, binodal and spinodal curves, together with critical or plait points. These geometrical elements define the boundaries of stable, metastable, and unstable domains of the ternary mixture in the phase diagram. While the experimental determination of the spinodal curve is intrinsically challenging, many techniques have been devised to obtain the binodal curve, from which a reliable model can then be derived to predict the location of the spinodal curve [1]. Still, the nature of the binodal curve is often ignored by experimentalists, who usually record the location of phase separation or solubilization. While correct from a phenomenological standpoint, half the information is then lost as the binodal curve is actually the combination of two branches of conjugated compositions, or nodes.

From an adequate experimental data set, the first computational problem is an inverse one. The thermodynamic potential must be derived from the experimental data. A vast literature treats the techniques to solve the inverse problem using different optimization algorithms, including stochastic optimization [2], genetic algorithms [3], the ant colony optimization method [4], and others. The most popular optimization criteria include the least-square minimization on the distance between the experimental and model estimated points and the tangent plane distance function, although other formulations are implemented in stochastic optimization algorithms [2]. We refer the interested reader to the review paper [5] for the detailed study of existing numerical approaches for parameter estimation in the phase equilibria context. We also remark that most of the published literature deals with NRTL or UNIQUAC models.

The second computational problem is a direct one. Both the binodal and spinodal curves should be computed over the whole composition range from the expression of the previously extracted expression of the thermodynamic potential. In order to compute the binodal curve, one needs to solve the set of algebraic equations expressing the equality between the chemical potentials in each phase for every component of the multicomponent mixture. Different methods are currently used to address this problem. The simplest one consists in solving these algebraic equations by a kind of Newton–Raphson iterative procedure over the discrete mesh approximating the state space [6]. Clearly, the accuracy of the result, as well as the computational effort is directly related to the finiteness of the mesh that is used. The famous liquid–liquid multi-phase flash algorithm proposed by Michelsen [7] uses the phase stability analysis based on the minimization of the distance between the tangent plane and the Gibbs energy surface, and hence explores the geometrical properties of the potential surface. Recently, Binous and Bellagi [8] proposed to use the arc-length continuation method, reducing the computation of the binodals to the integration of differential-algebraic (DEA) systems of equations with high accuracy. It is worth also citing the homotopic methods, see [9] and references therein, which allow solving simultaneously direct and indirect problems.

Interestingly, both computation problems are challenging due to the intrinsic geometry of the equilibrium curves that we briefly recapitulate. Since the pioneering works of D.J. Korteweg [10], the mathematical description of phase diagrams can be set in terms of topological properties of the surface associated with the appropriate thermodynamic potential, for instance, the Gibbs free energy *G*. The problem is rather simple in two-parametric systems (one-component biphasic system with varying temperature or pressure, bi-component systems under isobaric–isothermal conditions, etc.). In this case, the analysis reduces to the detection of the bitangent lines and the inflection points of the graph of the potential. However, in the ternary case, this picture becomes much more complicated.

Indeed, the binodal curves result from the projections of the one-parametric families of bitangent lines to the surface, while the spinodal curves are the projections of the curves of zero Gauss curvature. Starting from the works of Arnold [11] and Varchenko [12], the types of possible singularities associated with different types of thermodynamic potentials have been the subject of many studies; see, for instance, refs. [13,14,15] and references therein. Most of these works treated the binary systems with varying pressure or temperature, though the developed methods can be generalized to any type of diagram.

In this work, we propose an algorithm able to treat both direct and inverse problems by the integration of ordinary differential equations (ODE). This huge simplification rests on the reformulation of the mathematical problems in a space adequate to the intrinsic geometry of ternary phase diagrams. More precisely, our key idea is to reformulate the definition of the binodal curves in an extended 4D space by associating a proper configuration space to each phase of the system. To this end, the phase coexistence conditions are rewritten in geometrical terms involving the notions of bitangent planes and conodal pairs of points. The binodal curves are then shown to be the projections of the integral curve of a certain vector field in the extended space. The same approach applied in the 2D composition plane yields the vector field generating the spinodal curves.

Using the described geometrical construction, the numerical computation of binodal and spinodal curves can be performed via the standard integration of a system of ODE equations by any conventional ODE solver. From the numerical point of view, the proposed method implements a kind of a differential path-following algorithm. This method is here applied to detect the binodal and spinodal curves of ternary liquid mixture diagrams of types 0, I, and II, defined according to Treybal’s [16] classification. The proposed approach can be implemented with a variety of models for the thermodynamic potential, in particular, excess Gibbs free energy models like NRTL or UNIQUAC. We chose here the expression based on the analytical Flory–Huggins model, modified with a ternary cross-term that accounts for the oversimplifying hypotheses underlying the standard Flory–Huggins model. Interaction parameters were found through a non-linear optimization procedure associated with a non-standard criterion, which accounts for the intrinsic geometry of the binodal curve. This choice of model is not exclusive to the computation of the binodal and spinodal curves via ODE integration, but it has several remarkable advantages. First of all, the Flory–Huggins model provides a good representation of mixtures composed of molecules of different lengths, for instance, when one or more compound is a polymer, as suggested by [1]. On the other hand, the linearity of the Flory–Huggins expression of the Gibbs free energy of mixing with respect to unknown parameters facilitates the resolution of the inverse LLE problem. Indeed, some of the unknown parameters can be computed directly in the binary case, provided the miscibility gap is known experimentally. This fact leads to an important simplification of the fitting procedure in the ternary case for the diagrams of type I and II.

The paper is organized as follows. In Section 2, we recall how the phase separation conditions of a biphasic system maintained at thermodynamic equilibrium are related to the Gibbs free energy surface topology. In Section 3, we present the main conceptual result of the paper. The problem is considered in the extended 4D space having the structure of product space of two copies of 2D configuration space associated with each phase. It is shown that the phase coexistence conditions define a smooth curve in this space, referred to as the *generalized binodal curve*. Each point of this curve projects on a tie-line of the phase diagram. The binodal curve computation is then reduced to the numerical integration of a system of four differential equations. Apart from the special case of zero (or “island”)-type diagrams, the starting point of such integration can be found by solving at most three binary problems on the boundaries of the composition triangle. In Section 4, the developed approach is applied to the analysis of the series of examples of ternary mixtures, modelled by using the Flory–Huggins equation with an additional triple interaction term. We show that the novel computation method performs nicely, and our fitting results are of similar to better accuracy relative to other authors who have used NRTL or UNIQUAC models with parameters carried out with much more powerful optimization algorithms.

## 2. Phase Separation: From Thermodynamics to Geometry

### 2.1. Phase Coexistence in Multicomponent Mixtures at Thermodynamic Equilibrium

Consider an *N*-component system of volume *V* characterized by temperature *T*, pressure *P*, and entropy *S*. Denote by $n_{i}$ the number of moles of the *i*-th component and set $n=(n_{1},\ldots ,n_{N})$. By choosing *P*, *T*, and n as the coordinates of the thermodynamic state space, the physicochemical properties of the system as a whole can be described by the Gibbs free energy G(P,T,n). Being a homogeneous function of the first order with respect to $n_{i}$, *G* can be expressed as

(1) $$G\left(P,T,n\right)=\sum_{i=1}^{N}n_{i}\mu_{i}\left(P,T,n\right),$$

where by definition, $\mu_{i}=\frac{\partial G}{\partial n_{i}}$ is the chemical potential of *i*-th component. The fundamental Gibbs equation

(2) $$dG=-SdT+VdP+\sum_{i=1}^{N}\mu_{i}dn_{i}$$

describes the infinitesimal changes in the state of the system.

Consider an isolated system maintained at thermodynamic equilibrium without chemical reactions, and assume that its components coexist in two phases denoted by superscripts *I* and II. Then $G=G^{I}+G^{II}$. Equations (1) and (2) are valid for each phase, while the equilibrium condition dG=0 reads $dG^{I}+dG^{II}=0$. Moreover, since $n_{tot}=\sum_{i=1}^{N}n_{i}=const$ and $n_{i}=n_{i}^{I}+n_{i}^{II}=const$, it follows that $dn_{i}^{I}=-dn_{i}^{II}$. Then, the equilibrium assumption implies the equality between the pressure and the temperature in two phases: $T^{I}=T^{II}$, $P^{I}=P^{II}$, as well as the equality of chemical potentials of each component in both phases:

(3) $$\mu_{i}^{I}\left(P,T,n\right)=\mu_{i}^{II}\left(P,T,n\right).\;i=1,\ldots ,N$$

In the remaining part of this paper, only the isobaric–isothermic conditions will be considered, and thus, the dependence of *G* on *P* and *T* will be neglected. It is also worth remarking that two different thermodynamic models might be used for the expressions of Gibbs energy and chemical potentials of two phases, and other types of diagrams (liquid–solid, solid–solid, etc.) can be modelled in this way. The computational method discussed in this paper can be easily adopted to this case, but for the sake of simplicity, it is assumed that both phases of the system are described by one single model of *G*.

### 2.2. Phase Coexistence Conditions in Partial Molar Variables

Most of the thermodynamic models of real mixtures, as well as the available data of phase separation, are given in terms of either mole, volume or mass fractions of the components. So, for practical purposes, it is more convenient to express the phase coexistence conditions with respect to one of these sets of variables, for example, the mole fractions. This choice makes no restriction on the computations presented below, but in the case of volume or mass fraction, the specific molar Gibbs energy should be replaced by Gibbs energy per unit of volume or mass.

Let $g=G/n_{tot}$ and $x_{i}=\frac{n_{i}}{n_{tot}}$ denote, respectively, the molar free Gibbs energy and the mole fractions of the components of the mixture. Since $\sum_{i=1}^{N}x_{i}=1$, only N−1 of them can be used as independent variables. In what follows, we denote $x=(x_{1},\ldots ,x_{N-1})$, so that $x_{N}=1-x_{1}-\ldots -x_{N-1}$. In terms of molar variables, the first N−1 equilibrium conditions (3) are equivalent to the following relations:

(4) $$\frac{\partial g^{I}\left(x^{I}\right)}{\partial x_{i}^{I}}=\frac{\partial g^{II}\left(x^{II}\right)}{\partial x_{i}^{II}},\;i=1,\ldots ,N-1,$$

while the N-th condition (3) yields

(5) $$g^{I}\left(x^{I}\right)-g^{II}\left(x^{II}\right)-\sum_{i=1}^{N-1}\frac{\partial g^{I}\left(x^{I}\right)}{\partial x_{i}^{I}}\left(x_{i}^{I}-x_{i}^{II}\right)=0.$$

The interested reader can find the detailed mathematical derivation of these conditions in Appendix A.

**Figure 1:**
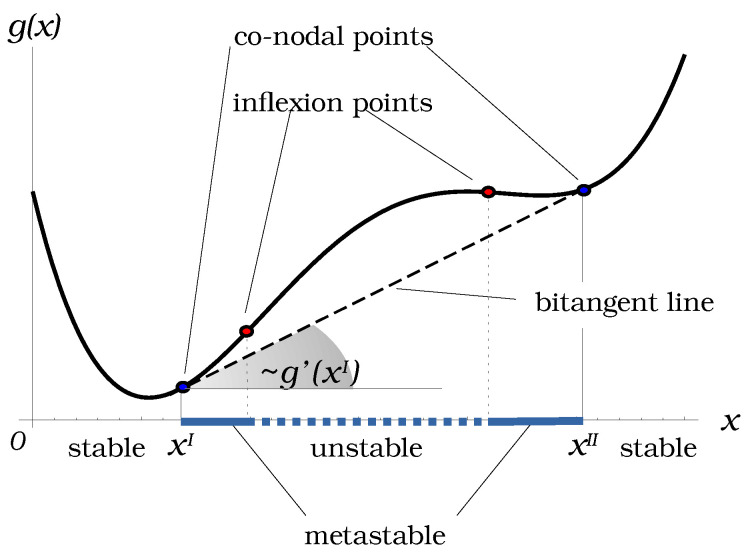
Two-phase separation in binary mixture: geometrical meaning of conditions (4) and (5).

The phase coexistence conditions written in forms (4) and (5) admit a clear geometrical interpretation. Indeed, in the particular case N=2, Equations (4) and (5) reduce to the conditions of existence of a bitangent line to the graph of the molar free energy function g(x), x∈[0,1]:

$$g^{\prime }\left(x^{I}\right)=g^{\prime }\left(x^{II}\right),\;g\left(x^{I}\right)-g\left(x^{II}\right)=g^{\prime }\left(x^{I}\right)\left(x^{II}-x^{I}\right),$$

as it is shown in Figure 1. Here, $g^{\prime }=\frac{\partial g}{\partial x}$. Other characteristics of the graph of *g*, like its convexity and the existence of inflexion points, are related to the material stability of the mixture. We will discuss them in a more general context in the next section.

### 2.3. Ternary Case: Phase Equilibrium Condition and Bitangent Planes Geometry

Let us now focus on a three-component liquid mixture whose components may coexist in one or two liquid phases. As in the binary case, in the ternary case, conditions (4) and (5) have a straightforward geometrical interpretation in terms of surface geometry.

Denote the composition domain of a ternary mixture by

$$\Omega \overset{\mathrm{def}}{=}\left\{x=\left(x_{1},x_{2}\right):\;x_{i}\in \left[0,1\right],\;i=1,2\;\mathrm{and}\;x_{1}+x_{2}\leq 1\right\}.$$

Consider a smooth surface

$$W\overset{\mathrm{def}}{=}\left\{\left(x,z\right)\in R^{3}:\;z=g\left(x\right),\;x\in \Omega \;\right\}$$

associated with the graph of the function g:Ω→R. The vector field

(6) $$N\left(x,z\right)=g_{1}^{\prime }\left(x\right)\partial_{x_{1}}+g_{2}^{\prime }\left(x\right)\partial_{x_{2}}-\partial_{z},$$

defines the normal direction to this surface. Here, $\partial_{x_{i}}$ are the coordinate vector fields in $R^{3}$ and $g_{i}^{\prime }=\frac{\partial g(x)}{\partial x_{i}}$, i=1,2. Let $P_{1}=\left(x^{I},g\left(x^{I}\right)\right)$ and $P_{2}=\left(x^{II},g\left(x^{II}\right)\right)$ be two points in $R^{3}$ belonging to the surface *W*, and such that

(7) $$g_{1}^{\prime }\left(x^{I}\right)=g_{1}^{\prime }\left(x^{II}\right),\;g_{2}^{\prime }\left(x^{I}\right)=g_{2}^{\prime }\left(x^{II}\right).$$

In view of (6), it means that the normals $N(P_{1})$ and $N(P_{2})$ are collinear.

Further, the vector $\bar{P_{1}P_{2}}=\left(x_{1}^{II}-x_{1}^{I},\;x_{2}^{II}-x_{2}^{I},\;g\left(x^{II}\right)-g\left(x^{I}\right)\right)$ belongs to the tangent plane $T_{P_{1}}W$ attached to the surface *W* at $P_{1}$, which means that $\bar{P_{1}P_{2}}⊥N\left(P_{1}\right)$, i.e.,

(8) $$g\left(x^{II}\right)-g\left(x^{I}\right)-g_{1}^{\prime }\left(x^{I}\right)\left(x_{1}^{II}-x_{1}^{I}\right)-g_{2}^{\prime }\left(x^{I}\right)\left(x_{2}^{II}-x_{2}^{I}\right)=0.$$

In view of condition (7), the vector $-\bar{P_{1}P_{2}}$ also belongs to $T_{P_{2}}W$. In other words, for N=3, conditions (4) and (5) mean that the surface *W* admits a bitangent plane passing through the points $P_{1}$ and $P_{2}$. Such pairs of points on the surface *W* are called conodal. It is easy to see that the projections on Ω of the points $P_{1}$ and $P_{2}$ along the *z*-axis define the compositions of splitting liquid phases $x^{I}$ and $x^{II}$.

The projection of the bitangent segment $P_{1}P_{2}$ on Ω is usually referred as node or tie-line. The curves on *W* formed by one-parametric families of conodal pairs define two directrices of a certain ruled surface in $R^{3}$, with the bitangent segments $P_{1}P_{2}$ being its generators. Projections of these directrices on Ω are called conodal or binodal curves of the phase diagram.

### 2.4. Differential Geometry of the Gibbs Energy Surface

As we have seen in the previous section, the geometry of the Gibbs energy surface *W* determines the topology of the underlying phase diagram. In fact, the most important properties of the phase diagram are encoded in the Gauss curvature of *W*. Denote by

$$H\left(x\right)\overset{\mathrm{def}}{=}{\left[\frac{\partial^{2}g\left(x\right)}{\partial x_{i}\partial x_{j}}\right]}_{i,j=1}^{N-1}$$

the Hessian associated to the function *g*. Then, the Gauss curvature of surface *W* takes the form [17]:

$$K\left(x\right)=\frac{\det H(x)}{{\left(g_{1}^{\prime }{\left(x\right)}^{2}+g_{2}^{\prime }{\left(x\right)}^{2}+1\right)}^{2}}$$

According to the sign of *K*, the composition domain Ω can partitioned into elliptic (K>0), parabolic (K=0), and hyperbolic (K<0) sub-domains. The surface *W* is also subdivided into elliptic and hyperbolic sub-domains by the parabolic curve {(x,z)∈W:K(x)=0}. On the other hand, *K* is a product of two principal curvatures of the surface: $K=k_{1}\cdot k_{2}$, so that the parabolic curve on *W* is the curve of zeros of one of the principal curvatures of the surface.

The vertical (i.e., along *z*-axis) projection of the parabolic curve on Ω defines the spinodal curve on the phase diagram. It bounds the elliptic sub-domain corresponding to the material stability domain of the mixture, which will be referred as $\tilde{\Omega }\overset{\mathrm{def}}{=}\left\{x\in \Omega :\;K\left(x\right)\geq 0\right\}$. It follows that the phase diagram can be described as the almost-Riemannian manifold $M=(\tilde{\Omega },H)$ with a border, equipped with an almost-Riemannian metric associated with the Hessian H(x). The sub-domain of $\tilde{\Omega }$ between the binodal and spinodal curves correspond to the metastable domain, while the remaining part delimited by the binodal curve and the boundary of Ω is the stable miscibility domain, where no phase separation occurs. In the binary case (see Figure 1), the binodal and spinodal curves reduce to two pairs of points that define the limits of stable and metastable domains.

The parabolic curves on smooth surfaces are very interesting geometrical objects. They may contain points where the curve has fourth-order contact with the tangent plane. Such special parabolic points (*godrons* in the terminology of [18]) correspond to the plait (critical) points of phase diagrams. It can be shown that special parabolic points give rise to two branches of conodal curves on *W*. In the underlying phase diagram, plait points are the only common points between spinodal and binodal curves. Besides plait points, binodal curves lie entirely in $\tilde{\Omega }$. The plait point location can be found by solving equations due to Tompa [19]:

(9) $$g_{222}^{\prime \prime \prime }{g_{11}^{\prime \prime }}^{2}-3g_{122}^{\prime \prime \prime }g_{11}^{\prime \prime }g_{12}^{\prime \prime }+3g_{112}^{\prime \prime \prime }{g_{12}^{\prime \prime }}^{2}-g_{111}^{\prime \prime \prime }g_{12}^{\prime \prime }g_{22}^{\prime \prime }=0,\;g_{11}^{\prime \prime }g_{22}^{\prime \prime }-{g_{12}^{\prime \prime }}^{2}=0$$

where $g_{ijk}^{\prime \prime \prime }=\frac{\partial^{3}g}{\partial x_{i}\partial x_{j}\partial x_{k}}$

**Figure 2:**
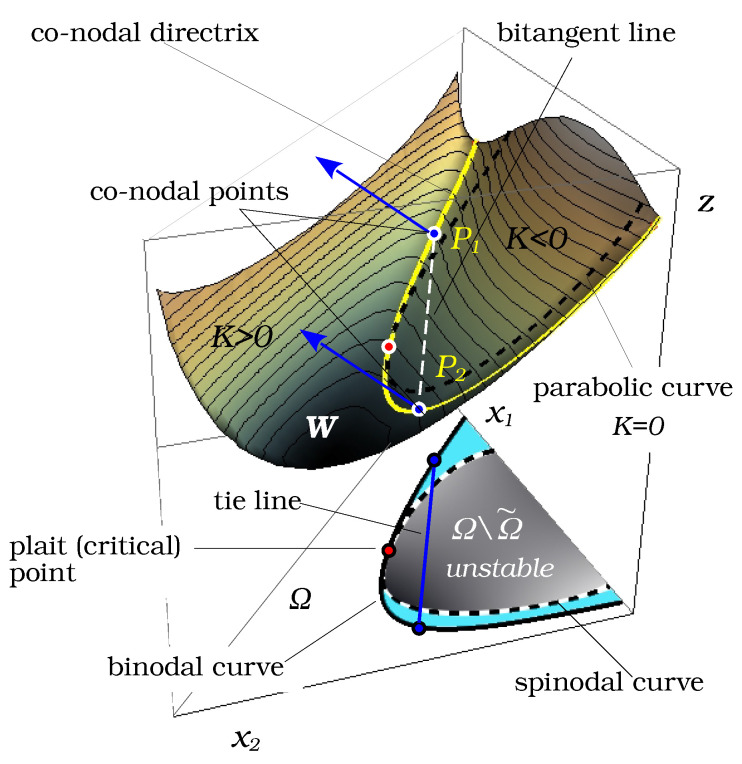
Gibbs energy surface *W* vs. the corresponding phase diagram. The hyperbolic domain of surface *W* is delimited by the parabolic curve (black dashed curve). Its vertical projection on Ω defines the spinodal curve (black and white dashed curve), which bounds the unstable domain of the phase diagram. The one-parametric family of conodal pairs of points on *W* (co-nodal directrix curve, yellow) projects on the binodal curve of the phase diagram. It can touch the spinodal curve at a plait point. The binodal curve divides the elliptic domain on Ω into stable (white) and metastable (light blue) sub-domains.

Figure 2 illustrates the described geometrical concepts.

## 3. Four-Dimensional Geometry of the Binodal Curve

So far, we have compared the 3D geometry of the surface *W* with the structure of the 2D phase diagram on Ω. But in order to better understand the intrinsic structure of the binodal curve, it would be more appropriate to associate a proper composition space $\Omega^{I}$ and $\Omega^{II}$ to each of the phases. Consider now an extended configuration space $\Sigma =\Omega^{I}\times \Omega^{II}$ of dimension four:

$$\Sigma \overset{\mathrm{def}}{=}\left\{q\in R^{4}\;:q=\left(q_{1},q_{2}\right)\;\mathrm{with}\;q_{1}=x^{I}\in \Omega^{I},\;q_{2}=x^{II}\in \Omega^{II}\right\}.$$

Equations (7) and (8) define three smooth sub-manifolds in Σ associated with zero-level sets of three functions $F_{i}:\Sigma \to R^{1}$ such that

(10) $$F_{1}\left(q\right)=g_{1}^{\prime }\left(q_{1}\right)-g_{1}^{\prime }\left(q_{2}\right),\;F_{2}\left(q\right)=g_{2}^{\prime }\left(q_{1}\right)-g_{2}^{\prime }\left(q_{2}\right),$$

$$F_{3}\left(q\right)=g\left(q_{2}\right)-g\left(q_{1}\right)+\left(\nabla_{x}g\left(q_{1}\right)|q_{2}-q_{1}\right),$$

where $\nabla_{x}g\left(q_{1}\right)=\left(g_{1}^{\prime }\left(q_{1}\right),g_{2}^{\prime }\left(q_{1}\right)\right)$ and (|) denote, respectively, the standard gradient and scalar product in $R^{2}$. The intersection of these 3D sub-manifolds defines a one-dimensional sub-manifold B⊂Σ such that

(11) $$B\overset{\mathrm{def}}{=}\left\{q\in \Sigma :\;F_{i}\left(q\right)=0,\;i=1,2,3\right\}.$$

The orthogonal projections $\pi_{i}:\Sigma \to \Omega$ such that $\pi_{I}\left(q\right)=q_{1}$ and $\pi_{II}\left(q\right)=q_{2}$ define two branches $\pi_{I}\left(B\right)$ and $\pi_{II}\left(B\right)$ of the binodal curve. In what follows, the sub-manifold *B* will be referred to as *the generalized binodal curve*.

Let $V\left(q\right)\in T_{q}B$ be the tangent vector at point q∈B of the generalized binodal *B* defined by (11). By construction, $V\in \overset{3}{\underset{i=1}{⋂}}Ker\nabla_{q}F_{i}$, so that by definition,

$$\nabla_{q}F_{i}\left(q\right)\cdot V\left(q\right)=0,\;i=1,2,3.$$

Computing the gradients of $F_{i}$ yields the following system of linear equations verified by components of the vector field $V=(V_{1},V_{2},V_{3},V_{4})$ at q∈B:

(12) $$\left(\begin{array}{cccc} g_{11}^{\prime \prime }\left(q_{1}\right) & g_{12}^{\prime \prime }\left(q_{1}\right) & -g_{11}^{\prime \prime }\left(q_{2}\right) & -g_{12}^{\prime \prime }\left(q_{2}\right)\\ g_{12}^{\prime \prime }\left(q_{1}\right) & g_{22}^{\prime \prime }\left(q_{1}\right) & -g_{12}^{\prime \prime }\left(q_{2}\right) & -g_{22}^{\prime \prime }\left(q_{2}\right)\\ \Phi_{1}\left(q\right) & \Psi_{1}\left(q\right) & 0 & 0 \end{array}\right)\cdot \left(\begin{array}{c} V_{1}\\ V_{2}\\ V_{3}\\ V_{4} \end{array}\right)=0,$$

where the functions $\Phi_{1}$ and $\Psi_{1}$ are defined by the relation

$$\left(\begin{array}{c} \Phi_{1}\\ \Psi_{1} \end{array}\right)=H\left(q_{1}\right)\cdot \left(q_{1}-q_{2}\right).$$

Rewriting (12) in a more compact form yields

(13) $$\Phi_{1}V_{1}+\Psi_{1}V_{2}=0,\;H\left(q_{2}\right)\left(\begin{array}{c} V_{3}\\ V_{4} \end{array}\right)=H\left(q_{1}\right)\left(\begin{array}{c} V_{1}\\ V_{2} \end{array}\right).$$

If $\det H(q_{2})\neq 0$ and at least one of the functions $\Phi_{1}$, $\Psi_{1}$ is non-zero, one obtains the solution of (13) in the form

$$V_{1}=\Psi_{1}\det H\left(q_{2}\right),\;V_{2}=-\Phi_{1}\det H\left(q_{2}\right)$$

and

$$\left(\begin{array}{c} V_{3}\\ V_{4} \end{array}\right)={\left(H(q_{2})\right)}^{-1}\cdot H\left(q_{1}\right)\cdot \left(\begin{array}{c} V_{1}\\ V_{2} \end{array}\right),$$

Performing all necessary simplifications, we obtain

(14) $$\left(\begin{array}{c} V_{1}\\ V_{2}\\ V_{3}\\ V_{4} \end{array}\right)=\left(\begin{array}{c} \Psi_{1}\det H\left(q_{2}\right)\\ -\Phi_{1}\det H\left(q_{2}\right)\\ \Psi_{2}\det H\left(q_{1}\right)\\ -\Phi_{2}\det H\left(q_{1}\right) \end{array}\right)$$

where, by definition,

(15) $$\left(\begin{array}{c} \Phi_{2}\\ \Psi_{2} \end{array}\right)=H\left(q_{2}\right)\cdot \left(q_{1}-q_{2}\right).$$

**Figure 3:**
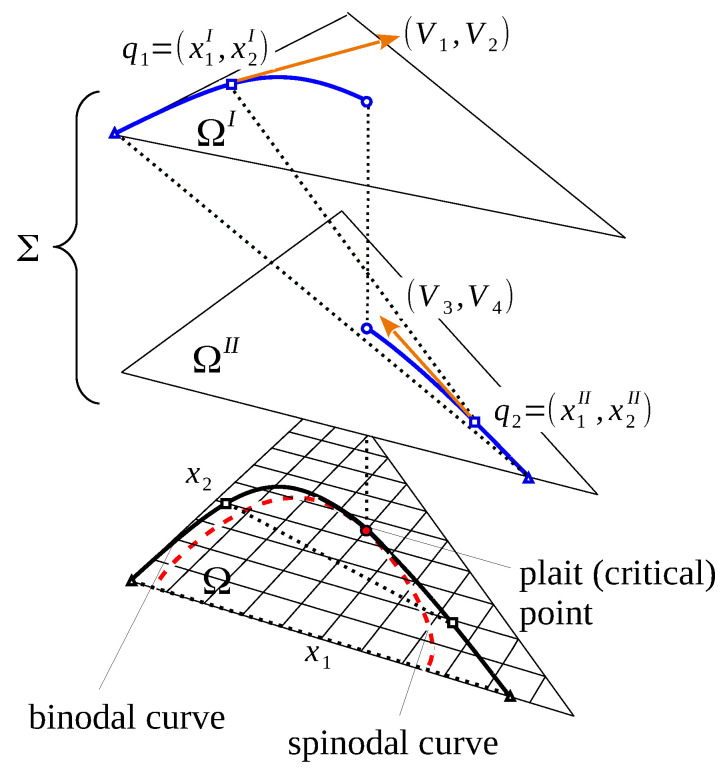
Four-dimensional lifting of the binodal curve. The complete configuration space of the binodal curve is $\Sigma =\Omega^{I}\times \Omega^{II}$. The vector field $V=(V_{1},V_{2},V_{3},V_{4})$ generates a smooth curve *B* (blue) in Σ. The orthogonal projections of *B* onto Ω form the binodal curve (black) on the underlying phase diagram. The dotted lines indicate the coupled pairs of phases, and the dashed red curve indicates the location of the spinodal curve.

The above expressions define a smooth vector field V∈TΣ. It is well defined except for the singular points q such that $\det H\left(q_{1}\right)=\det H\left(q_{2}\right)=0$, in other words, if $q_{1}$ and $q_{2}$ belong to the spinodal, i.e., if they coincide forming the plait point of the phase diagram. The described geometrical construction is shown in Figure 3.

Summing up, we showed that the binodal curve can be easily recovered from the projection of the integral curve of the vector field *V*. Moreover, the particular structure of formula (14), namely the properties (13) and (15), imply that the tie-lines are orthogonal to the binodal with respect the metric in $\tilde{\Omega }$ associated with the Hessian matrix H(x).

### Numerical Computation of Binodal and Spinodal Curves

Being the integral curve of the vector field *V*, the generalized binodal curve can be computed by solving the system of ordinary differential equations $\dot{q}=V\left(q\right)$ in Σ. Here, the dot notation stands for the derivative with respect to any suitable parameter. This result has an important practical application regarding its computation.

Using the vector field *V*, the numerical computation of binodal curves reduces to a simple ODE integration by any conventional solver with desired accuracy. The normalization of *V* allows one to avoid the eventual stiffness problem when approaching the border of $\tilde{\Omega }$, so it is recommended to use the arc-length parameter for the integration. To start the computation, one needs to find an initial point in Σ, i.e., a starting tie-line of the binodal curve. This can be performed by analysing the profile of the *W* surface along the boundaries of the composition domain Ω, in other words, by solving at most three binary problems over the interval [0,1]. The only exception here are the closed-type 0 phase diagrams, for which the initial tie-line must be found inside Ω.

The same method can be applied to derive the differential equation of the spinodal curve. This case is even simpler because it is a problem in the 2D space Ω. Indeed, being the solution of the equation H(x)=0, the spinodal curve is a solution to the differential equation associated with the vector field $S\left(x\right)=\nabla_{x}H{\left(x\right)}^{⊥}$ in Ω, which, in general, is regular at plait points. Here, the superscript ${}^{⊥}$ denotes the orthogonal complement to the vector $\nabla_{x}H\left(x\right)$ in $R^{2}$. In other words, the spinodal curve is the solution of the ODE equation $\dot{x}=S\left(x\right)$, x∈Ω; the starting point for the integration can be detected by finding one inflexion point of the function *g* on the boundary of Ω or inside Ω for closed-type curves.

Once binodal and spinodal curves are computed, the plait point can be found by solving numerically Equation (9), taking the approximate common point between these curves as the initial guess for the non-linear solver in order to facilitate the converge procedure.

The implementation of the described computational method requires accessing the derivatives of the thermodynamic model defining the function *g* up to the order two. The finite-difference method may be not sufficient to obtain the necessary accuracy level for the Hessian computation. In fact, this is the main obstacle in the implementation of such types of algorithms in many other domains involving the thermodynamic models of real systems. However, we would like to stress the fact that the nowadays numerical technology allows one to easily overcome this inconvenience. For the academic use, the symbolic computation packages, like Maple or Mathematica, which we used in this paper, would be more than sufficient. To develop a stand-alone calculator of phase diagram, the automatic differentiation technique can be employed. A pilot version of a functional code of this kind was described in [20] for the computation of the univolatility curves of the residue curve maps.

## 4. Inverse Problem and Case Studies

Following [16], ternary LLE phase diagrams of biphasic systems can be roughly divided into three main classes depending on the number of the partially miscible binary pairs:

- Type 0 or “island” type: The diagram is characterized by a closed heterogeneous domain inside the composition triangle, while all three binary pairs are miscible. The systems of this type exhibit two plait points.
- Type I: One pair of components exhibits a miscibility gap on the border of the composition triangle. This type of diagram possesses one plait point where both liquid phases have the same composition. This is the most common type of phase diagrams (75% according to [21]).
- Type II: This type is characterized by the presence of two partial miscibility gaps on the borders of the composition triangle. Such diagrams do not have plait points. They represent about 20% of known solutions ([21]).

Under variations in temperature or pressure, all these types of behaviour transform one into another, or split into two or more heterogeneous zones. Two heterogeneous zones can also melt, forming three phase domains. The analysis of this phenomenon goes beyond the aim of this paper. The three case studies presented below correspond to the three standard types of diagrams, using the Flory–Huggins model for the free energy function *g*.

### 4.1. The Flory–Huggins Model Equation

The choice of an appropriate thermodynamic model for function *g* depends on the particular application. For LLE diagrams, NRTL or UNIQIUAC models that describe a huge class of ternary systems, were studied by numerous authors, though the quality of the model parameter regression is not always satisfactory. As suggested by [1], for the polymer solutions, the Flory–Huggins (FH) model can be employed to describe the systems of type non-solvent–solvent–polymer, solvent–polymer–polymer, and even three polymers. The relative simplicity of the mathematical expression of constitutive equation is the main advantage of the FH model with respect to NRTL or UNIQUAC models used by other authors. Unlike NRTL or UNIQUAC, formulated in terms of mole fractions, the FH model uses partial volume fractions, assuming that the total volume of the systems is equal to the sum of partial volumes.

The classical Flory–Huggins model defines the free energy of mixing per unit of volume according to the expression

(16) $$g=\frac{x_{1}}{N1}\ln x_{1}+\frac{x_{2}}{N_{2}}\ln x_{2}+\frac{x_{3}}{N_{3}}\ln x_{3}+\chi_{12}x_{1}x_{2}+\chi_{13}x_{1}x_{3}+\chi_{23}x_{2}x_{3}+\beta x_{1}x_{2}x_{3}.$$

Here $x_{i}$, i=1,2,3 are the volume fractions of the components, and $N_{2}$ and $N_{3}$ and relative numbers of segments in the molecules of the compounds with respect to the first compound, which usually corresponds to water; thus, $N_{1}=1$. The symbols $\chi_{ij}$ and β stay for the binary and ternary interaction coefficients. In all computation below, $x_{3}$ was replaced by $x_{3}=1-x_{1}-x_{2}$.

In most of the papers that use Equation (16), the ternary coefficient β=0, while some of the binary interaction coefficients may depend on the components of the composition vector *x*. However, the triple interaction term accounts for the possible linear dependency of $\chi_{ij}$ on *x*, and allows one to relax the total volume conservation hypothesis when mixing the components. So, in this paper, to maintain the model equations as simple as possible, the six parameters $N_{i}$, $\chi_{ij}$, and β are assumed to be constant. In other words, in this paper, we propose considering (16) as a formal mathematical expression without taking into account the exact physical meaning of its parameters, at least in the cases where constant volume assumption is difficult to justify.

### 4.2. Parameter Estimation Procedure and Case Studies

In order to calculate the LLE diagrams presented below, a set of $n_{b}$ tie-line measurements was used.

In order to fit the model, the six parameters of the FH model were computed via a non-linear optimization procedure, associated with the function

(17) $$\min_{p}\left(\sum_{k=1}^{n_{b}}\frac{F_{1}^{2}\left(q_{k}\right)+F_{2}^{2}\left(q_{k}\right)+F_{3}^{2}\left(q_{k}\right)}{{\left(1-F_{3}^{2}\left(q_{k}\right)\right)}^{2}}\right),$$

where $F_{i}$ are defined by (10), and *p* is the vector of unknown scalar parameters. The denominator term penalizes those pairs of points that do not belong to the same bitangent line. In our examples this criterion showed a better convergence to the experimental data comparing to the sole square term in the nominator of (17). The exact mathematical expressions of functions $F_{i}$ associated with the FH model are provided in Appendix B.1.

**Figure 4:**
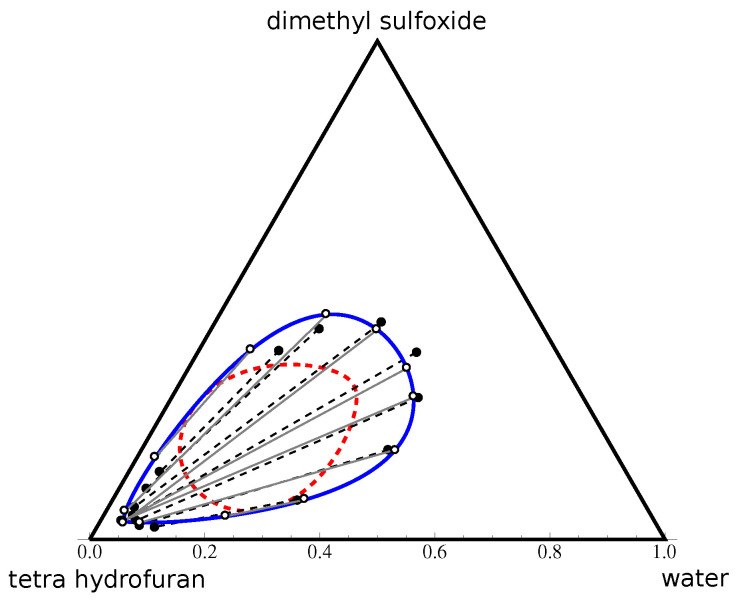
Phase diagram of water–DMSO–THF in the volume fraction space. Here, black points and dashed lines correspond to the experimental points and tie-lines from [23]. The blue curve and the red dashed curve represent, respectively, the binodal and spinodal curves calculated using the Flory–Huggins model. White points indicate the end-points of the FH-calculated tie-lines (grey) used in the RMSD evaluation.

**Figure 5:**
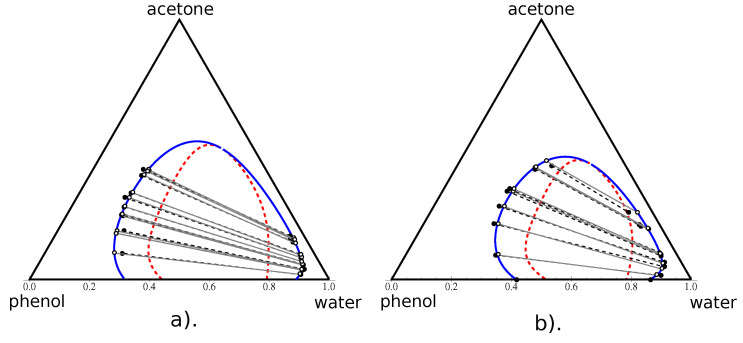
Phase diagram of water–phenol–acetone in the volume fraction space at 50 °C (**a**) and at 56 °C (**b**). Black points and dashed lines correspond to the experimental points and tie-lines from [26] (left) and [23]. The blue curve and the red dashed curve represent, respectively, the binodal and spinodal curves calculated using the Flory–Huggins model. White points indicate the end-points of the FH-calculated tie-lines (grey) used in the RMSD evaluation.

The described NLP minimization procedure was applied to compute two of the examples below: the type 0 diagram water–dimethyl sulfoxide (DMSO)–tetra hydrofuran (THF) (see in Figure 4) and water–phenol–acetone at 50 °C (Figure 5a). However, for the diagrams of types I and II, the computation can be simplified, provided the measurement of at least one of binary miscibility gaps is available. In fact, in this case, the linearity of the FH model with respect to the parameters allows one to compute the part of the parameters describing the binary pair straightforwardly, and thus reduces the number of unknowns of problem (17). The computation formulae are provided in Appendix B.1. Finally, the quality of the predicted model can be evaluated using the standard formula for the root mean square deviation (RMSD)

$$\sigma =100{\left(\frac{1}{6n}\sum_{k=1}^{n_{b}}\sum_{i}^{3}{\left(x_{i,k}^{I,FH}-x_{i,k}^{I,exp}\right)}^{2}+{\left(x_{i,k}^{II,FH}-x_{i,k}^{II,exp}\right)}^{2}\right)}^{1/2}$$

used by other authors.

All numerical computations presented in this paper were realized by Mathematica 9 software [22]. We used the standard functions with the default choice of methods. Namely, the ODE integration was performed by NDSolve function, which implements the LSODA algorithm (combined Adams and BDF methods). The optimization problem was solved by the FindMinimum command with the quasi-Newton method chosen by default. The original unpublished data used for the LLE regression are included in Appendix C, together with the computational formulae for the Flory–Huggins model available in Appendix B.1.

#### 4.2.1. Type 0 Diagram: Water–DMSO–THF

The parametric regression of type 0 diagrams is known to be a difficult task. Figure 4 represents our result of the phase diagram prediction based on the experimental data available in [23] for the water–DMSO–THF system. These data were also studied in [8,24,25] using NRTL and UNIQUAC models. In Table 1, the computed RMSD value of the reconstructed FH model is compared with those obtained by other authors, showing a good quality of prediction for this mixture using the FH model.

#### 4.2.2. Type I Diagram: Water–Phenol–Acetone

The ternary diagrams of the water–phenol–acetone system at 50 °C and 60 °C using NRTL and UNIQUAC models were studied in [24,26], both using the same data from [26]. Our result of the FH regression according the method described above is shown in Figure 5a). In Figure 5b, we show the phase diagram for 56 °C computed using the data from [23]. In this second case, the computation was simplified because [23] provides the measurement on the phenol–water miscibility gap. It allows one to compute explicitly the values of $\chi_{13}$ and $N_{3}$. The remaining four parameters were estimated via the general optimization procedure described above. Again, the comparison with the RMSD values published by other authors shows a very good quality of our result for 50 °C, and the best one for 56 °C.

#### 4.2.3. Type II Diagram: Water–Acetone–Hexadecane

**Figure 6:**
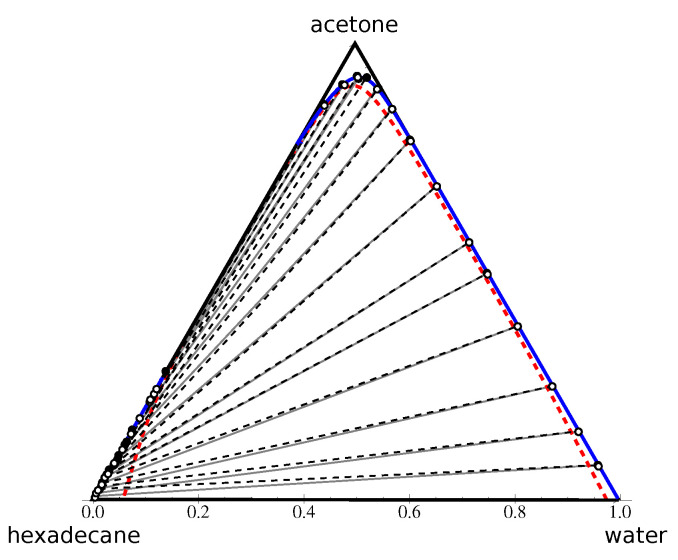
Water–acetone–hexadecane system in the volume fraction space. Black points and dashed lines correspond to the experimental points and tie-lines. The blue curve and the red dashed curve represent, respectively, the binodal and spinodal curves calculated using the Flory–Huggins model. White points indicate the end-points of the FH-calculated tie-lines (grey) used in the RMSD evaluation.

For the water–acetone–hexadecane system, we used our own set of 14 tie-line measurements obtained with Raman’s spectroscopy. Another set of 17 measurements of the phase envelope, without taking into account the conodal pair correspondence, were obtained using the redisolution by adding acetone. More details on the experimental procedure are available in [27]; the data are included in Appendix C, Table A1 and Table A2. The phase envelope measurements allowed one to detect the acetone–hexadecane miscibility gap. For the miscibility gap of water–hexadecane, we used the data accessible in the literature. Knowing these two miscibility gaps, we first computed the four parameters $N_{2}$, $N_{3}$, $\chi_{13}$, and $\chi_{23}$. Due to the significant variation of the solubility data of hexadecane in water in different sources, the term $\chi_{13}$ was replaced by the $\chi_{13}+\delta$, where the correction term δ was adjusted in the next step of computation. After this important simplification, the number of unknown parameters was reduced to three. Applying the optimization protocol on the remaining set of parameters produced the result depicted in Figure 6.

This result shows an excellent prediction for shape of phase envelope, and a very good quality for the tie-lines’ directions. The RMSD value associated with this predictions equal to 2.92.

## 5. Conclusions

This work showed that the challenging computation of liquid–liquid phase diagrams can be drastically simplified by taking into account the intrinsic geometrical structures associated with phase equilibrium conditions. The crucial mathematical idea is to work in an adequate extended space. Indeed, we show that the stability domain boundary defined by the 2D binodal curve is a projection of a higher-dimensional object, the 4D generalized binodal curve, which can be easily computed by solving a set of ordinary differential equations.

This mathematical viewpoint is employed to propose a new numerical algorithm for phase-diagram computation, which drastically reduces the computation effort and guarantees a high value of accuracy. Notably, the only iterative procedure is the one used to find the initial point for the integration. We tested this methodology on four ternary liquid phase diagrams of different topological types. We chose a modified Flory–Huggins model for the regression procedure applied to the available experimental data. The presented results show a good, sometimes excellent, accordance between the data and the calculated model, which opens a promising perspective for the further development of the method to study other types of multiphase diagrams. Furthermore, the developed approach can be used for any other type of ternary diagram and is valid for any chosen thermodynamic model.

## Figures and Tables

**Table 1:** Root mean square deviation σ of analysed mixture.

System	Model	T, *C*	σ
water–DMSO–THF	FH (this work)	20°	3.53
water–DMSO–THF, [25]	NRTL	20°	5.83
water–DMSO–THF, [8]	UNIQUAC	20°	3.4
water–DMSO–THF, [24]	NRTL	20°	3.18, 3.09
water–DMSO–THF, [24]	UNIQUAC	20°	3.97, 3.4
water–acetone–phenol	FH (this work)	50°	0.88
water–acetone–phenol, [26]	NRTL	50°	0.81
water–acetone–phenol, [24]	NRTL	50°	1.13
water–acetone–phenol, [24]	UNIQIAC	50°	1.17
water–acetone–phenol	FH (this work)	56°	1.27
water–acetone–phenol, [23]	NRTL	56°	1.61
water–acetone–phenol, [23]	UNIQUAC	56°	1.52
water–acetone–hexadecane	FH (this work)	20°	2.92

## Data Availability

The original experimental data are included in Appendix C.

## Appendix A. Phase Coexistence Conditions in Terms of Molar Fractions

Let $g=G/n_{tot}$ denote the molar free Gibbs energy. Since the mole fractions $x_{i}=\frac{n_{i}}{n_{tot}}$ verify $\sum_{i=1}^{N}x_{i}=1$, only N−1 of them can be used as independent variables. In what follows, we denote $x=(x_{1},\ldots ,x_{N-1})$, so that $n_{N}=n_{tot}\left(1-x_{1}-...-x_{N-1}\right)$. Since $dx_{N}=-\sum_{i=1}^{N-1}dx_{i}$, the Gibbs Equation (2) takes the form

(A1) $$dg=-sdT+vdP+\sum_{i=1}^{N-1}\left(\mu_{i}-\mu_{N}\right)dx_{i},$$

where $s=S/n_{tot}$ and $v=V/n_{tot}$ are the specific molar entropy and volume, correspondingly. Moreover, for all i=1,…,N−1 we obtain

(A2) $$\frac{\partial g}{\partial x_{i}}=\frac{1}{n}\frac{\partial G}{\partial x_{i}}=\frac{1}{n}\left(\frac{\partial G}{\partial n_{i}}\frac{\partial n_{i}}{\partial x_{i}}+\frac{\partial G}{\partial n_{N}}\frac{\partial n_{N}}{\partial x_{i}}\right)=\mu_{i}-\mu_{N},$$

and hence,

(A3) $$g=\frac{1}{n}\sum_{i=1}^{N}\mu_{i}n_{i}=\sum_{i=1}^{N-1}\left(\mu_{i}-\mu_{N}\right)x_{i}+\mu_{N}.$$

Rewriting (A2), (A3) for each phase, the phase coexistence Equations (3) can be expressed in terms of mole fractions. Indeed, the first N−1 equilibrium conditions (3) are equivalent to the following relations:

(A4) $$\frac{\partial g^{I}\left(x^{I}\right)}{\partial x_{i}^{I}}=\frac{\partial g^{II}\left(x^{II}\right)}{\partial x_{i}^{II}},\;i=1,\ldots ,N-1,$$

while the N-th condition (3) yields

(A5) $$g^{I}\left(x^{I}\right)-g^{II}\left(x^{II}\right)-\sum_{i=1}^{N-1}\frac{\partial g^{I}\left(x^{I}\right)}{\partial x_{i}^{I}}\left(x_{i}^{I}-x_{i}^{II}\right)=0.$$

## Appendix B. The Flory–Huggins Model Computational Formulae

### Appendix B.1. Phase Coexistence Conditions Associated with Flory–Huggins Model and Linearity

In the case of the Flory-Huggins model (16) the mathematical expressions of functions $F_{i}$ take the forms

$$F_{1}=\chi_{12}\left(x_{2}^{I}-x_{2}^{II}\right)-\chi_{13}\left(2\left(x_{1}^{I}-x_{1}^{II}\right)+x_{2}^{I}-x_{2}^{II}\right)-\chi_{23}\left(x_{2}^{I}-x_{2}^{II}\right)$$

$$+\beta \left(x_{2}^{I}-2x_{1}^{I}x_{2}^{I}-{\left(x_{2}^{I}\right)}^{2}-x_{2}^{II}+2x_{1}^{II}x_{2}^{II}+{\left(x_{2}^{II}\right)}^{2}\right)$$

$$+\frac{1}{N_{1}}\ln\left(\frac{x_{1}^{I}}{x_{1}^{II}}\right)-\frac{1}{N_{3}}\ln\left(\frac{1-x_{1}^{I}-x_{2}^{I}}{1-x_{1}^{I}-x_{2}^{II}}\right)$$

$$F_{2}=\chi_{12}\left(x_{1}^{I}-x_{1}^{II}\right)-\chi_{13}\left(x_{1}^{I}-x_{1}^{II}\right)-\chi_{23}\left(x_{1}^{I}-x_{1}^{II}+2x_{2}^{I}-2x_{2}^{II}\right)+$$

$$+\beta \left(x_{1}^{I}-{\left(x_{1}^{I}\right)}^{2}-x_{1}^{II}+{\left(x_{1}^{II}\right)}^{2}-2x_{1}^{I}x_{2}^{I}+2x_{1}^{II}x_{2}^{II}\right)$$

(A6) $$+\frac{1}{N_{2}}\ln\left(\frac{x_{2}^{I}}{x_{2}^{II}}\right)-\frac{1}{N_{3}}\ln\left(\frac{1-x_{1}^{I}-x_{2}^{I}}{1-x_{1}^{II}-x_{2}^{II}}\right)$$

$$F_{3}=\chi_{12}\left(x_{1}^{I}-x_{1}^{II}\right)\left(x_{2}^{I}-x_{2}^{II}\right)-\chi_{13}\left(x_{1}^{I}-x_{1}^{II}\right)\left(x_{1}^{I}-x_{1}^{II}+x_{2}^{I}-x_{2}^{II}\right)$$

$$-\chi_{23}\left(x_{2}^{I}-x_{2}^{II}\right)\left(x_{1}^{I}-x_{1}^{II}+x_{2}^{I}-x_{2}^{II}\right)$$

$$+\beta \left(x_{1}^{I}x_{2}^{I}-2{\left(x_{1}^{I}\right)}^{2}x_{2}^{I}-x_{1}^{II}x_{2}^{I}+2x_{1}^{I}x_{1}^{II}x_{2}^{I}-2x_{1}^{I}{\left(x_{2}^{I}\right)}^{2}+x_{1}^{II}{\left(x_{2}^{I}\right)}^{2}\right.$$

$$\left.-x_{1}^{I}x_{2}^{II}+{\left(x_{1}^{I}\right)}^{2}x_{2}^{II}+x_{1}^{II}x_{2}^{II}-{\left(x_{1}^{II}\right)}^{2}x_{2}^{II}+2x_{1}^{I}x_{2}^{I}x_{2}^{II}-x_{1}^{II}{\left(x_{2}^{II}\right)}^{2}\right)$$

$$+\frac{1}{N_{1}}\left(x_{1}^{I}-x_{1}^{II}-x_{1}^{II}\ln\left(\frac{x_{1}^{I}}{x_{1}^{II}}\right)\right)+\frac{1}{N_{2}}\left(x_{2}^{I}-x_{2}^{II}-x_{2}^{II}\ln\left(\frac{x_{1}^{I}}{x_{1}^{II}}\right)\right)$$

$$-\frac{1}{N_{3}}\left(x_{1}^{I}-x_{1}^{II}+x_{2}^{I}-x_{2}^{II}-\left(1-x_{1}^{II}-x_{2}^{II}\right)\ln\left(\frac{1-x_{1}^{I}-x_{2}^{I}}{1-x_{1}^{II}-x_{2}^{II}}\right)\right)$$

The phase coexistence conditions (4) and (5) take the form $F_{i}\left(q\right)=0$ for i=1,2,3 and $q=(x_{1}^{I},x_{2}^{I},x_{1}^{II},x_{2}^{II})\in \Sigma$. Remarkably, for the FH model, these conditions are linear with respect to the interaction parameters $\chi_{ij}$, β and to the $1/N_{2}$ and $1/N_{3}$ values. This fact permits the direct computation of these constants in the binary case, as it is explained in the next section.

### Appendix B.2. Detection of the Parameters of the Fh Model from the Miscibility Gap Limits

Assume that components 1 and 3 are partially miscible and assume that the values $x_{1}^{I}$ and $x_{1}^{II}$ are available through the experimental procedure. The binary mixture of components 1–3 corresponds to the side $x_{2}=0$ of the boundary of Ω. Setting $x_{1}=x$, $x_{3}=1-x$, the function *g* can be rewritten in the form

$$g_{1}=\frac{x}{N_{1}}\ln x+\frac{1-x}{N_{3}}\ln\left(1-x\right)+\chi_{13}x\left(1-x\right).$$

Using expressions (A6), the phase coexistence conditions (4) and (5) applied to the function $g_{1}$ then have the form

(A7) $$2\left(x^{I}-x^{II}\right)\chi_{13}+\frac{1}{N_{3}}\ln\left(\frac{1-x^{I}}{1-x^{II}}\right)-\ln\left(\frac{x^{I}}{x^{II}}\right)=0,$$

(A8) $$x^{I}-x^{II}-\chi_{13}{\left(x^{I}-x^{II}\right)}^{2}+\left(1-x^{II}\right)\ln\left(\frac{1-x^{I}}{1-x^{II}}\right)$$

$$-\frac{1}{N_{3}}\left(x^{I}-x^{II}-x^{II}\ln\left(\frac{x^{I}}{x^{II}}\right)\right)=0.$$

If the values of $x^{I}$ and $x^{II}$ are known, the above equations form a system of two linear algebraic equations with respect to $\chi_{13}$ and $1/N_{3}$, which can be easily computed explicitly.

Assume that both couples 1–3 and 2–3 are partially miscible. Then, the same procedure as above can be applied to find parameters $N_{2}$, $N_{3}$, $\chi_{13}$, and $\chi_{23}$ by solving a system of four linear algebraic equations in terms of the miscibility gap limits $x_{1}^{I},x_{1}^{II}$ and $x_{2}^{I},x_{2}^{II}$.

## Appendix C. Water–Acetone–Hexadecane Experimental Data

**Table A1:** Water–acetone–hexadecane tie-lines, mass fraction experimental data.

Phase I			Phase II		
**Water**	**Acetone**	**Hexadecane**	**Water**	**Acetone**	**Hexadecane**
0.005026	0.088051	0.906923	0.1153287	0.88	0.0046713
0.002956	0.121763	0.875281	0.07389669	0.9115	0.014603313
0.004319	0.127764	0.867917	0.05061999	0.919	0.030380015
0.000775	0.153273	0.845952	0.02604692	0.9075	0.06645308
0.000313	0.284572	0.715115	0.01003813	0.865	0.124961871
0.002187	0.023547	0.974265	0.93897	0.06102	0.000000001
0.002148	0.029435	0.968417	0.87777	0.12223	0.000000001
0.001949	0.036801	0.96125	0.793004	0.20699	0.000000001
0.000308	0.048658	0.951034	0.67413	0.3259	0.000000001
0.003093	0.054403	0.942503	0.56358	0.4364	0.000000001
0.000276	0.065321	0.934403	0.49548	0.50452	0.000000001
0.005298	0.074969	0.919733	0.3655	0.63446	0.000000001
0.003066	0.082343	0.914591	0.2541	0.74585	0.000000001
0.002307	0.097121	0.900573	0.1747	0.82524	0.000000001

**Table A2:** Water–acetone–hexadecane phase envelope, mass fraction experimental data. The highlighted values correspond to the miscibility gap of the binary mixture acetone–hexadecane.

Water	Acetone	Hexadecane
0.3503	0.6496	8.98 × 10^−5^
0.1989	0.7996	0.0015
0.1519	0.846	0.002
0.115	0.882	0.0033
0.10056065	0.89269389	0.00674546
0.06122825	0.91649876	0.02227299
0.04112357	0.9178071	0.04106932
0.02740997	0.90841079	0.06417925
0.01445356	0.88430202	0.10124442
0.0039	0.8456	0.1504
0.002	0.799	0.199
**0**	**0.783**	**0.217**
**0**	**0.264**	**0.736**
0.0005	0.1975	0.8021
0.0007	0.1589	0.8404
0.00092	0.1122	0.8869
0.00065	0.0603	0.9391
